# Supplementary material for: Magnetic stimulation allows focal activation of the mouse cochlea
Source: eLife. 2022 May 24;11:e76682. doi: 10.7554/eLife.76682 (PMC9177144; doi:10.7554/eLife.76682)
Supplement: Transparent reporting form [file elife-76682-transrepform1.pdf]

eLIFE
1st Floor
24 Hills Road
Cambridge CB2 1JP, UK
P 01223 855340
W elifesciences.org
T @elife

eLife's transparent reporting form

We encourage authors to provide detailed information *within their submission* to facilitate the interpretation and replication of experiments. Authors can upload supporting documentation to indicate the use of appropriate reporting guidelines for health-related research (see EQUATOR Network), life science research (see the BioSharing Information Resource), or the ARRIVE guidelines for reporting work involving animal research. Where applicable, authors should refer to any relevant reporting standards documents in this form.

If you have any questions, please consult our Journal Policies and/or contact us: editorial@elifesciences.org.

Sample-size estimation

- You should state whether an appropriate sample size was computed when the study was being designed
- You should state the statistical method of sample size computation and any required assumptions
- If no explicit power analysis was used, you should describe how you decided what sample (replicate) size (number) to use

Please outline where this information can be found within the submission (e.g., sections or figure legends), or explain why this information doesn't apply to your submission:

The sample size was determined based on our previous (substantial) experience with magnetic stimulation of other types of CNS sensory neurons, and is consistent with previous investigations with cochlear implants. Based on these norms, we acquired data from at least six animals for each modality. Capturing responses to apical stimulation proved more challenging (n = 3 animals) but still support our key findings from stimulation of basal cochlea. In addition, the second set of control experimetns involving gentamicin-treated mice (n = 2) were limited to only 2 animals. They provide strong confirmation of our principal control experiment in which DI water was used to destroy cochlear function. Responses to each stimulus condition were recorded at least 39 times, which has been adequate to demonstrate statistical significance. This information is detailed in Results each time a new set of data or analysis is introduced, as well as in the stimulation section of Materials and Methods.

Replicates

- You should report how often each experiment was performed
- You should include a definition of biological versus technical replication
- The data obtained should be provided and sufficient information should be provided to indicate the number of independent biological and/or technical replicates
- If you encountered any outliers, you should describe how these were handled
- Criteria for exclusion/inclusion of data should be clearly stated
- High-throughput sequence data should be uploaded before submission, with a private link for reviewers provided (these are available from both GEO and ArrayExpress)

Please outline where this information can be found within the submission (e.g., sections or figure legends), or explain why this information doesn't apply to your submission:

eLife Sciences Publications, Ltd is a limited liability non-profit non-stock corporation incorporated in the State of Delaware, USA, with company number 5030732, and is registered in the UK with company number FC030576 and branch number BR015634 at the address 1st Floor, 24 Hills Road, Cambridge CB2 1JP | August 2014

Each modality was tested with at least six animals (biological replicates). At least three individual coils/electrodes with an identical design were tested (technical replicates). Immunostaining from two individual animals was performed (biological replicates). Replicate information is included in the corresponding sections of Results, the figure legends, and the magnetic and electric stimulation section of Materials and Methods. Animals without acoustic responses were excluded from experiments. Again, all of this is detailed in the manuscript.

Statistical reporting

- Statistical analysis methods should be described and justified
- Raw data should be presented in figures whenever informative to do so (typically when N per group is less than 10)
- For each experiment, you should identify the statistical tests used, exact values of N, definitions of center, methods of multiple test correction, and dispersion and precision measures (e.g., mean, median, SD, SEM, confidence intervals; and, for the major substantive results, a measure of effect size (e.g., Pearson's r, Cohen's d)
- Report exact p-values wherever possible alongside the summary statistics and 95% confidence intervals. These should be reported for all key questions and not only when the p-value is less than 0.05.

Please outline where this information can be found within the submission (e.g., sections or figure legends), or explain why this information doesn't apply to your submission:

We provided statistical information each time significance was assessed for an experiment – details are in the text and/or the figure legends. All statistical tests are described in the statistical analysis section of Materials and Methods. P values for analysis of group data are reported to at least three significant digits.

(For large datasets, or papers with a very large number of statistical tests, you may upload a single table file with tests, Ns, etc., with reference to sections in the manuscript.)

Group allocation

- Indicate how samples were allocated into experimental groups (in the case of clinical studies, please specify allocation to treatment method); if randomization was used, please also state if restricted randomization was applied
- Indicate if masking was used during group allocation, data collection and/or data analysis

Please outline where this information can be found within the submission (e.g., sections or figure legends), or explain why this information doesn't apply to your submission:

Whenever possible, all modalities were presented on the same animal. Random grouping or masking was not needed in our experiments.

Additional data files ("source data")

- We encourage you to upload relevant additional data files, such as numerical data that are represented as a graph in a figure, or as a summary table
- Where provided, these should be in the most useful format, and they can be uploaded as "Source data" files linked to a main figure or table
- Include model definition files including the full list of parameters used

- Include code used for data analysis (e.g., R, MatLab)
- Avoid stating that data files are “available upon request”

Please indicate the figures or tables for which source data files have been provided:

The source data and codes are available on the Open Science Framework (DOI 10.17605/OSF.IO/Y7ZRX).
